# Professional Self‐Perception Among Critical Care Nurses in China: A Nationwide Cross‐Sectional Survey

**DOI:** 10.1155/jonm/6657778

**Published:** 2026-03-13

**Authors:** Xin Liu, Xuan Zhang, Zewen Pan, Yu Xu, Zhongwen Sun, Haixia Tang, Lei Chen, Jianhua Tang, Zheng Jia, Yue Huang, Ying Peng, Hanfeng Zhang, Zhenjun Liu

**Affiliations:** ^1^ Department of Critical Care Medicine, Sichuan Clinical Research Center for Cancer, Sichuan Cancer Hospital & Institute, Sichuan Cancer Center, University of Electronic Science and Technology of China, Chengdu, China, uestc.edu.cn; ^2^ Department of Colorectal Surgery, Sichuan Clinical Research Center for Cancer, Sichuan Cancer Hospital & Institute, Sichuan Cancer Center, University of Electronic Science and Technology of China, Chengdu, China, uestc.edu.cn; ^3^ Department of Critical Care Medicine, West China Hospital, West China School of Nursing, Sichuan University, Chengdu, Sichuan, China, scu.edu.cn; ^4^ Department of Critical Care Medicine, State Key Laboratory of Oncology in South China, Guangdong Provincial Clinical Research Center for Cancer, Sun Yat-sen University Cancer Center, Guangzhou, China, sysucc.org.cn; ^5^ Department of Critical Care Medicine, Traditional Chinese Hospital of Lu’an Affiliated to Anhui University of Traditional Chinese Medicine, Lu’an, Anhui, China; ^6^ Department of Critical Care Medicine, National Clinical Research Center for Cancer, Tianjin’s Clinical Research Center for Cancer, Tianjin Medical University Cancer Institute and Hospital, Tianjin, China, tmucih.com; ^7^ Department of General Internal Medicine, Sichuan Clinical Research Center for Cancer, Sichuan Cancer Hospital & Institute, Sichuan Cancer Center, University of Electronic Science and Technology of China, Chengdu, Sichuan, China, uestc.edu.cn; ^8^ Department of Nursing, Sichuan Clinical Research Center for Cancer, Sichuan Cancer Hospital & Institute, Sichuan Cancer Center, University of Electronic Science and Technology of China, Chengdu, China, uestc.edu.cn

**Keywords:** adjustment focus, intensive care unit, professional self-perception, thriving at work, voice behavior

## Abstract

**Objective:**

This study aimed to conduct a nationwide cross‐sectional investigation to gain a comprehensive understanding of the professional self‐perception among critical care nurses in China. This investigation focused on three dimensions: the Thriving at Work Scale (TWS), the Voice Behavior Scale (VBS), and the Adjustment Focus Scale (AFS).

**Method:**

An electronic questionnaire was distributed to intensive care unit (ICU) nurses across China using stratified and convenience sampling. TWS, VBS, and AFS scores and sociodemographic factors were collected. Multivariable ordinal logistic regression (MOLR) was employed to explore the influencing factors on professional self‐perception (tertiled TWS, VBS, and AFS scores).

**Result:**

A total of 583 valid questionnaires were collected. The TWS, VBS, and AFS scores were 49 (44–54), 37 (31–40), and 48 (45–55), respectively. MOLR analysis revealed significant associations between gender and TWS (adjusted OR, 95% CI: 2.01, 1.32–3.07, *p* < 0.001), VBS (adjusted OR, 95% CI: 2.15, 1.39–3.33, *p* < 0.001), and AFS (adjusted OR, 95% CI: 1.74, 1.14–2.66, *p* = 0.002). Average monthly household income was significantly associated with TWS (adjusted OR, 95% CI: 1.51, 1.19–1.92, *p* < 0.001), VBS (adjusted OR, 95% CI: 1.35, 1.05–1.74, *p* = 0.018), and AFS (adjusted OR, 95% CI: 1.47, 1.16–1.86, *p* = 0.002). Professional title was inversely related to TWS (adjusted OR, 95% CI: 0.73, 0.55–0.96, *p* = 0.027) and VBS (adjusted OR, 95% CI: 0.64, 0.47–0.88, *p* = 0.006). In addition, associations were found between marital status and TWS, ICU experience and position and VBS, and age and AFS (*p* < 0.05).

**Conclusion:**

Our findings suggest that critical care nurses in China exhibit a moderate level of professional self‐perception. Young, single, female ICU nurses with lower household income, middle‐level professional titles, lower positions, and less ICU experience are associated with lower professional self‐perception. Nursing managers can intervene to enhance professional self‐perception by targeting individuals with these influencing factors.

## 1. Introduction

The concept of nursing professional self‐perception​ encompasses the subjective cognitions and feelings that nurses experience regarding their role, competence, attitudes, beliefs, motivations, and career satisfaction [1, 2]. This perception is influenced by various factors such as education, expectations, experiences, and the social and cultural context within which they work [3].

A positive nursing professional self‐perception is associated with higher levels of self‐esteem, job satisfaction, and enhanced patient care. Nurses with a strong sense of professional self‐perception tend to view their work as valuable and meaningful, leading to increased commitment to providing high‐quality care. Conversely, a negative nursing professional self‐perception can result in low job satisfaction, high turnover rates, and unfavorable patient outcomes [4, 5]. Nurses who feel undervalued or unappreciated may lack the motivation to offer optimal care and are more likely to experience burnout, stress, and ultimately leave their jobs [4]. Overall, nursing professionals′ self‐perception is a crucial aspect of nursing practice that plays a critical role in shaping the quality of care provided and the well‐being of nurses themselves.

The studies targeting to improve nursing professionals′ self‐perception are scanty. Only two research studies indicated that education and training improved professional self‐perception of nurses [1, 6]. Furthermore, the nursing professional self‐perception is a complex and multifaceted construct [5, 7]. Professional self‐perception among nurses refers to how they view themselves within a professional context, which is dynamic, context‐sensitive, and focused on situational judgments of one’s professional identity and performance. Nursing professional self‐perception involves self‐assessment of professional competence (mastery of clinical skills, critical thinking, and evidence‐based practice), perception of professional role and value (contribution, autonomy, responsibility, and recognition within the profession), and sensitivity to external feedback and context (adjustment in self‐perception based on social evaluations, work environment, and cultural or systemic factors) [7, 8]. Thriving at work is a holistic psychological state; high levels of thriving are associated with reduced burnout, improved job performance, better health, and fostered self‐growth and professional development [9]. Thriving (vitality and learning) could be framed as an outcome of basic psychological needs satisfaction, which influences self‐perception. Nurses who perceive themselves positively are more likely to thrive at work, as they feel confident in their abilities and value their contributions [10]. Voice behavior is the tendency of employees to express their opinions, ideas, and concerns to management or colleagues [11]. Nurses with a strong professional self‐perception are inclined to exhibit voice behavior because they feel empowered to speak up and believe their input is valuable [11, 12]. In addition, a positive professional self‐perception influences a nurse’s adjustment focus. When nurses hold their profession in high regard, they are more likely to adopt a prevention focus, emphasizing safety and error avoidance while also seeking new learning opportunities and striving for excellence, which can lead to an increased promotion focus [13, 14]. Accordingly, thriving at work reflects competence and relatedness, voice behavior expresses responsibility and autonomy, and adjustment focus aligns with goal‐oriented self‐regulation, all contributing to a multifaceted professional self‐perception.

Moreover, due to high work intensity and mental stress, including burnout and emotional exhaustion [15], intensive care unit (ICU) nurses experience significant turnover rates and a global shortage [16]. A shortage of critical care staff can potentially lead to increased mortality among critically ill patients [17]. Cultivating a strong professional self‐perception is crucial for enhancing the quality of care, improving patient outcomes, and reducing nurse turnover. However, high clinical stress and work intensity may negatively impact professional self‐perception. In addition, Chinese culture has some unique characteristics, such as collectivism and hierarchical order [18, 19], which may profoundly impact the professional self‐perception of nurses. At present, no specific scales target the assessment of critical care nurses’ professional self‐perception in China.

Therefore, our objective was to assess the professional self‐perception of Chinese ICU nurses using the three dimensions of Thriving at Work Scale (TWS), Voice Behavior Scale (VBS), and Adjustment Focus Scale (AFS) as proxies. In addition, we aimed to explore significant sociodemographic factors influencing the professional self‐perception of this unique population. Related research remains limited in the existing nursing literature.

## 2. Methods

### 2.1. Settings

This nationwide cross‐sectional study was conducted from August to September 2024 and was approved by the Ethics Committee for Medical Research of Sichuan Cancer Hospital (SCCHEC‐02‐2024‐216). Participants were informed about the confidentiality and anonymity of their responses, detailed on the first page of an online questionnaire. Basic information; TWS, VBS, and AFS scores; and sociodemographic variables were collected. Full consents were obtained from all participants.

### 2.2. Participants

The study enrolled ICU nurses from tertiary hospitals nationwide. Stratified and convenience sampling were employed to cover hospitals in five geographical regions of mainland China (western, northern, southern, eastern, and central). We randomly selected four provinces in each region (totaling 20 provinces) and then utilized convenience sampling to contact potential ICU nursing heads or managers for online questionnaire distribution.

Inclusion criteria were as follows: (a) a minimum of 1 year of experience working in an ICU and (b) willingness to participate in this study. Exclusion criteria included any absence from ICU work for more than 3 months due to pregnancy, illness, or family matters.

### 2.3. Measurements

#### 2.3.1. TWS

The TWS developed by Porath et al. [20] comprises two dimensions: vitality and learning, each consisting of five items. Items are rated on a 7‐point Likert scale, ranging from “completely inconsistent” to “completely consistent,” yielding total scores between 10 and 70. In this study, the Cronbach’s alpha coefficient for the TWS was 0.829.

#### 2.3.2. VBS

The VBS, developed by Liang et al. [21], comprises two dimensions: promotive suggestion behavior and inhibitive suggestion behavior, each consisting of five items. Items are rated on a 5‐point Likert scale ranging from “strongly disagree” to “strongly agree,” yielding scores between 10 and 50. The overall Cronbach’s alpha coefficient for the VBS in this study was 0.949.

#### 2.3.3. AFS

The AFS, based on Higgins’ regulatory focus theory by Wallace et al. [14], was translated and revised by Chinese scholars for nurses [22]. The scale consists of 12 items divided into two dimensions: promotive focus (6 items) and preventive focus (6 items). It employs a 5‐point Likert scale, similar to the VBS, with total scores ranging from 12 to 60. The Cronbach’s alpha coefficient for the AFS in this study was 0.947.

### 2.4. Data Collection

An online survey platform, WenjuanXing, was used to collect all information. Prior to survey distribution, two trained investigators distributed the survey, explained details of each item, and responded to questions from participating ICU nursing heads or managers via telephone, email, and WeChat (a popular social application in China). Before initiating the survey, the study’s objectives and informed consent were presented. Collected information was checked by the main researchers and two investigators for logical errors and duplicate submissions. Surveys with missing items could not be submitted.

### 2.5. Statistical Analysis

The statistical analysis was performed using R (R Foundation for Statistical Computing Platform, Version 4.4.0). Quantitative variables were presented as mean ± standard deviation or median with interquartile range (25^th^–75^th^ percentile). Pearson correlation analysis was used to explore the relationship between TWS, VBS, and AFS. Comparisons between groups were conducted using the Wilcoxon or Kruskal–Wallis rank sum test after nonparametric distribution was confirmed by the Kolmogorov–Smirnov test. Bonferroni correction is used for multiple comparisons. Categorical variables were expressed as frequencies (percentages).

A multivariable ordinal logistic regression (MOLR) was employed to investigate the influencing factors and facilitate convenient interpretation of results, considering the non‐normal dependent variables and categorical independent variables (sociodemographic variables). The proportional odds assumption (POA) for the MOLR was tested using the Brant test, which assesses whether the effect of independent variables on the cumulative probability remains consistent across all categories of these ordinal outcomes (tertiled TWS, VBS, and AFS scores). Two steps of MOLR were adopted. During the first step, significant variables were identified; in the second step, different levels of these significant variables were compared.

The minimum sample size required for this study was estimated to be 20 times the number of independent variables [23], resulting in a total of 280 participants due to the inclusion of 14 independent variables. Significant variables were selected using a backward selection method based on the Akaike information criterion (AIC). All *p* values were calculated as two‐sided tests, and *p* < 0.05 was considered statistically significant.

## 3. Results

### 3.1. Participants’ Characteristics

A total of 600 questionnaires were distributed, of which 583 were valid, yielding a 97.2% response rate. The study enrolled ICU nurses from 23 hospitals across western, northern, southern, eastern, and central China (Supporting Figure S1). The study population predominantly comprised females (83.2%), individuals with bachelor’s degrees (81.1%), clinical nurses (80.8%), contractually employed individuals (66.7%), nurses with average monthly incomes of 5001–10,000 yuan (approximately $700–$1400, 56.1%), and those with average monthly household incomes of 10,001–30,000 yuan (approximately $1401–$4201, 52%). The majority held the professional title of senior nurse (50.1%) or nurse in charge (31.7%). More detailed characteristics are presented in Table 1.

**TABLE 1:** Sociodemographic characteristics of the participants.

Variable	*N*	%
Age (years)		
20–29	256	43.9
30–39	275	47.2
≥ 40	52	8.9
Gender		
Male	98	16.8
Female	485	83.2
Marital status		
Single	203	34.8
Married	372	64.8
Divorced or widowed	8	1.4
Fertility status		
Infertile	264	45.3
Fertile	319	54.7
Education level		
Specialized education	98	16.8
Bachelor’s degree	473	81.1
Master’s degree and above	12	1.2
Professional title		
Nurse	81	13.9
Senior nurse	292	50.1
Nurse in charge	185	31.7
Deputy chief nurse and above	25	4.3
Position		
Clinical nurse	471	80.8
Group lead nurse	58	9.9
Head nurse	54	9.3
Employment type		
Formal	98	16.8
Personnel agency	70	12.0
Contractual	389	66.7
Labor dispatch	26	4.5
ICU experience (years)		
1–5	249	42.7
6–10	160	27.4
11–15	125	21.4
> 15	49	8.4
Average monthly income		
≤ 5000 yuan (≤ $700)	137	23.5
5001–10000 yuan ($700–$1400)	327	56.1
10,001–15,000 yuan ($1401–$2101)	73	12.5
> 15,000 yuan (> $2101)	46	7.9
Average monthly household income		
≤ 10,000 yuan (≤ $1400)	231	39.6
10,001–30,000 yuan ($1401–$4201)	303	52.0
30,001–50,000 yuan ($4202–$7003)	39	6.7
> 50,000 yuan (> $7003)	10	1.7
Nurse specialist training		
No	323	55.4
Yes	260	44.6
Weekly working hours		
≤ 40	137	23.5
41–50	368	63.1
51–60	46	7.9
> 60	32	5.5

### 3.2. Scores and Correlation of Thriving at Work, Voice Behavior, and Adjustment Focus

The TWS, VBS, and AFS scores were 49 (44–54), 37 (31–40), and 48 (45–55), respectively. As depicted in Figure 1(a), the distributions exhibit long left tails and wide ranges, particularly for VBS. The correlation matrix in Figure 1(b) demonstrates significant positive correlations between TWS, VBS, and AFS (all *p* < 0.001).7

FIGURE 1Distribution and correlation of TWS, VBS, and AFS. (a) Distribution of TWS, VBS, and AFS. (b) Correlation matrix of TWS, VBS, and AFS. TWS: Thriving at Work Scale; VBS: Voice Behavior Scale; AFS: Adjustment Focus Scale.

**Figure:**
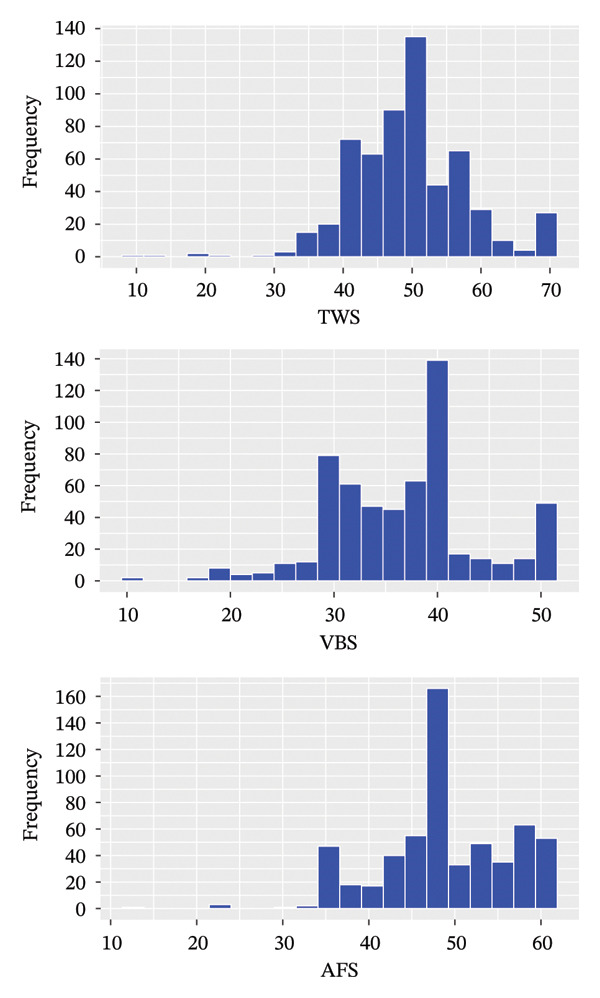
(a)

**Figure:**
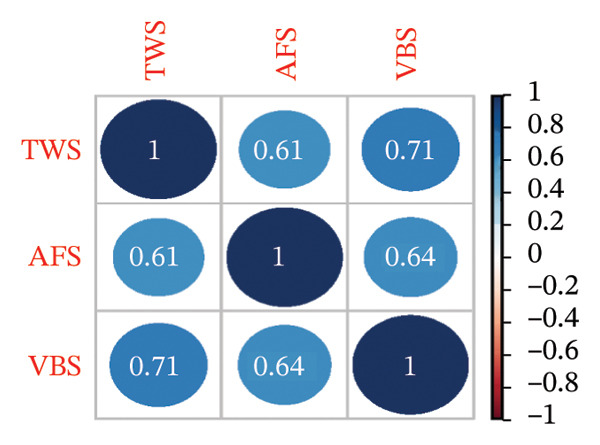
(b)

### 3.3. Factors Associated With Thriving at Work, Voice Behavior, and Adjustment Focus by Univariable and MOLR Analysis

In the univariable analysis, significant differences were observed in terms of gender, marital status, average monthly income, and average monthly household income with regard to TWS, VBS, and AFS (all *p* < 0.05). Detailed results with significant comparison differences are depicted in Figure 2. Interestingly, education level and weekly working hours did not significantly differ (both *p* > 0.05). Variables without significant comparison differences are illustrated in Supporting Figure 2.

FIGURE 2Comparison of significant sociodemographic factors related to professional self‐perception in the violin plots. (a) TWS. (b) VBS. (c) AFS. TWS: Thriving at Work Scale; VBS: Voice Behavior Scale; AFS: Adjustment Focus Scale. The wider parts of the violin represent areas where the data are more densely concentrated, the line inside the box represents the median value, and the box represents the interquartile range. The whiskers extend from the box and represent the range of the data; outliers are represented with solid dots in whiskers, and subtransparent dots represent data distribution with some jitters. ^∗^ *p* < 0.05, ^∗∗^ *p* < 0.01, and ^∗∗∗^ *p* < 0.001.

**Figure:**
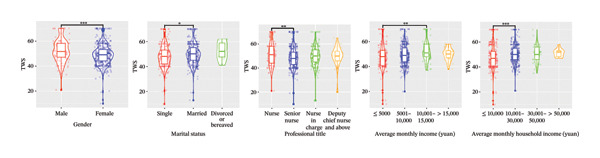
(a)

**Figure:**
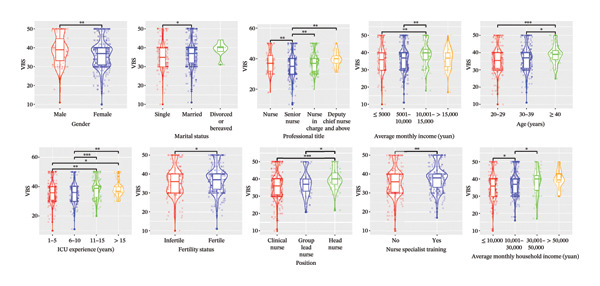
(b)

**Figure:**
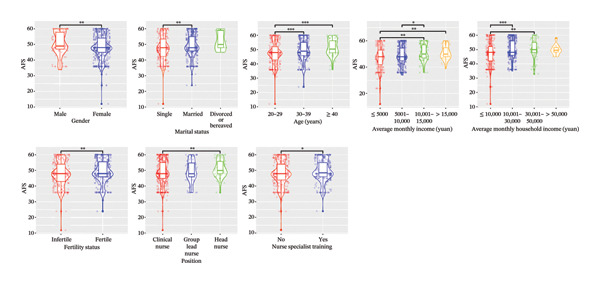
(c)

No violation of the POA was found for TWS (*p* = 0.27), VBS (*p* = 0.34), or AFS (*p* = 0.75) in the Brant test of MOLR analysis. MOLR analysis revealed a significant association between gender and thriving at work (*p* < 0.001), voice behavior (*p* < 0.001), and adjustment focus (*p* = 0.002), as shown in Table 2.

**TABLE 2:** Factors associated with scores on the TWS, VBS, and AFS by ordinal logistic analysis.

Variables	TWS			VBS			AFS		
	AOR	95% CI	*p* value	AOR	95% CI	*p* value	AOR	95% CI	*p* value
Age							2.04	1.37–3.06	< 0.001
Gender	2.01	1.32–3.07	< 0.001	2.15	1.39–3.33	< 0.001	1.74	1.14–2.66	0.002
ICU experience				1.28	1.04–1.59	0.021			
AMHI	1.51	1.19–1.92	< 0.001	1.35	1.05–1.74	0.018	1.47	1.16–1.86	0.002
Marital status	1.52	1.06–2.19	0.022						
Professional title	0.73	0.55–0.96	0.027	0.64	0.47–0.88	0.006			
Position				1.63	1.22–2.18	0.001			

Abbreviations: AFS, Adjustment Focus Scale; AMHI, average monthly household income; AOR, adjusted odds ratio; CI, confidence interval; TWS, Thriving at Work Scale; VBS, Voice Behavior Scale.

Average monthly household income was found to be a significant factor influencing thriving at work (*p* < 0.001), voice behavior (*p* = 0.018), and adjustment focus (*p* = 0.002). Interestingly, professional title was inversely associated with TWS (*p* = 0.027) and VBS (*p* = 0.006).

In addition, marital status was independently associated with thriving at work (*p* = 0.022). ICU experience (*p* = 0.021) and position (*p* = 0.001) were significantly associated with voice behavior. Age was independently associated with adjustment focus (*p* = 0.022). No interactional effects were observed. Supporting Information should contain word descriptions presenting results of univariable and multivariable analyses.

### 3.4. Various Levels of Variables Related to Thriving at Work, Voice Behavior, and Adjustment Focus Analyzed by MOLR

Our study indicated that male nurses were 2.10 times more likely to have higher TWS scores (adjusted OR, 95% CI: 2.10, 1.39–3.19, *p* < 0.001), 2.01 times more likely to have higher VBS scores (adjusted OR, 95% CI: 2.01, 1.31–3.10, *p* = 0.001), and 1.92 times more likely to have higher AFS scores (adjusted OR, 95% CI: 1.92, 1.27–2.92, *p* = 0.002) compared to female nurses, as determined by MOLR analysis adjusted for significant variables. These results are summarized in Figure 3.

FIGURE 3The association between various levels of sociodemographic factors and professional self‐perception of ICU nurses by MOLR analysis. MOLR: multivariable ordinal logistic regression; TWS: Thriving at Work Scale; VBS: Voice Behavior Scale; AFS: Adjustment Focus Scale; AOR: adjusted odds ratio; AMHI: average monthly household income.

**Figure:**
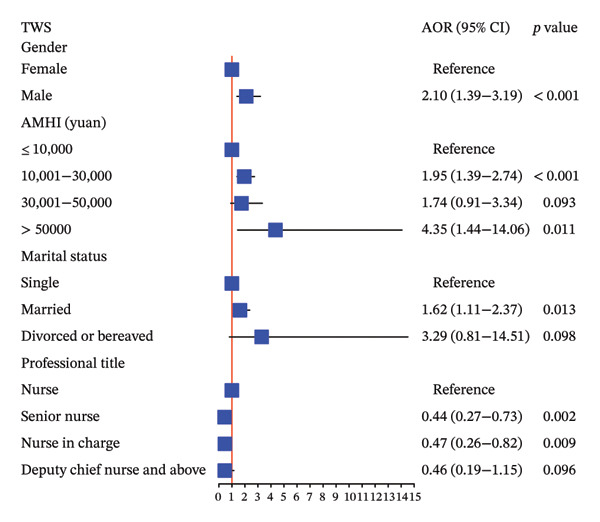
(a)

**Figure:**
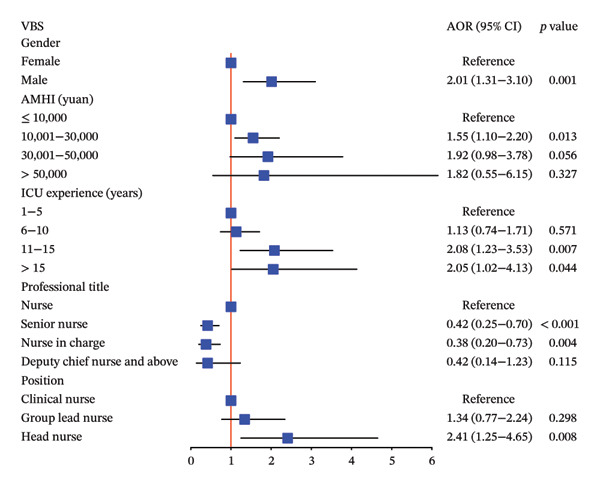
(b)

**Figure:**
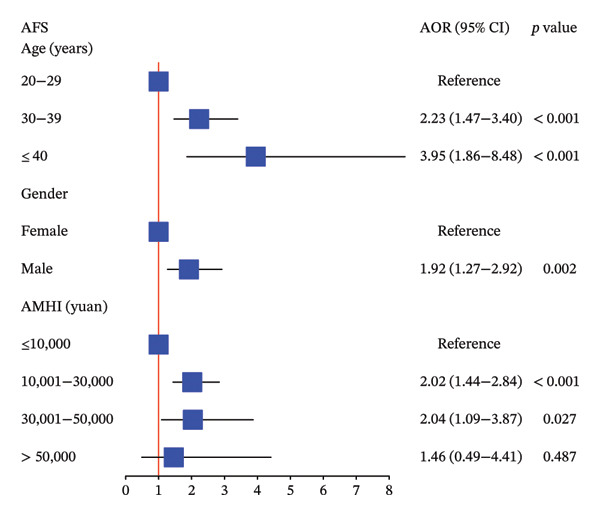
(c)

Furthermore, higher average monthly household income was associated with increased TWS, VBS, and AFS scores, although statistical significance was not observed at each level, as shown in Figure 3. Notably, nurses with higher professional titles exhibited an association with lower TWS, VBS, and AFS scores. Specifically, compared to regular nurses, senior nurses were associated with significantly decreased thriving at work (adjusted OR, 95% CI: 0.44, 0.27–0.73, *p* = 0.002) and voice behavior (adjusted OR, 95% CI: 0.42, 0.25–0.70, *p* < 0.001). Similar results were observed for nurses in charge, with significantly decreased thriving at work (adjusted OR, 95% CI: 0.47, 0.26–0.82, *p* = 0.009) and voice behavior (adjusted OR, 95% CI: 0.38, 0.20–0.73, *p* = 0.004).

Married nurses demonstrated significantly higher TWS scores compared to single nurses (adjusted OR, 95% CI: 1.62, 1.11–2.37, *p* = 0.013). In addition, nurses with extensive ICU experience and higher positions tended to engage in voice behavior, although statistical significance was not reached at some levels. Aged ICU nurses (≥ 40 years) exhibited 2.23‐fold and 3.95‐fold increased AFS scores compared to their middle‐aged (30–39 years) and younger counterparts (20–29 years), respectively (each *p* < 0.001).

### 3.5. Sensitivity Analysis of Gender Differences in Professional Self‐Perception

Given the gender distribution imbalance (485 female nurses, 83.2% of the total sample), potential bias could be introduced into the assessment of gender differences in thriving at work, voice behavior, and adjustment focus. To mitigate this, a sensitivity analysis was performed using gender‐balanced random resampling of our data (98 female and 98 male nurses, 50% each). Consistent statistically significant gender differences were observed in TWS, VBS, and AFS scores (*p* = 0.011, *p* = 0.039, and *p* = 0.024, respectively), as illustrated in Supporting Figure 3.

## 4. Discussion

This study investigated the professional self‐perception of ICU nurses by examining three dimensions: thriving at work, voice behavior, and adjustment focus. Thriving at work is a critical psychological driver of individual growth and development [24], positively impacting mental health, reducing stress levels, preventing burnout, and fostering self‐leadership [25, 26]. At the individual level, voice behavior is associated with alleviating job stress, optimizing the work environment, and enhancing job performance of nurses [12, 27]. Adjustment focus (or regulatory focus) reflects a nurse’s pursuit of positive results, proactive exploration, and self‐improvement, while avoiding negative results and prioritizing safety precautions [13].

Furthermore, thriving at work, voice behavior, and adjustment focus are interconnected. Thriving at work can promote nurses’ voice behavior [28], contributing to their sense of validation and continuous learning [29]. There is also a reciprocal relationship between thriving at work and adjustment focus [30], along with a positive correlation between voice behavior and adjustment focus [31]. Correlation analyses in our study supported these interconnections, demonstrating statistical significance. Thus, we assessed professional self‐perception among nurses using the three interconnected dimensions of thriving at work, voice behavior, and adjustment focus. A positive professional self‐perception can enhance job satisfaction, commitment, and performance [4].

In our study, the TWS scores for ICU nurses averaged 49 (range: 44–54), comparable to emergency department nurses (51.06 ± 7.77) [32]. The VBS and AFS scores of ICU nurses were 37 (31–40) and 48 (45–55), respectively, consistent with previous studies involving multidisciplinary nurses with VBS scores (35.1 ± 8.1) [33] and AFS scores (49.31 ± 6.93) [34]. Overall, TWS, VBS, and AFS scores of ICU nurses in China were moderate, although some nurses exhibited notably low scores (as evidenced by the left tail of histograms in Figure 1 and the low position of outlines and whiskers in violin plots in Figure 2), indicating a poor professional self‐perception. Identifying key influencing factors is essential for implementing interventions to prevent adverse outcomes such as errors or turnover.

We found that gender, average monthly household income, professional title, marital status, and age were identified as the primary factors influencing nurses’ professional self‐perception. Both univariable and multivariable analyses revealed that male ICU nurses had higher professional self‐perception, as evidenced by greater TWS, VBS, and AFS scores compared to female ICU nurses. Our findings contradict those of a single‐center study in Egypt, which reported lower TWS scores among male critical care nurses [35]. A study involving emergency department nurses in China also reported elevated TWS scores in males than females, although significance was not achieved [32]. The differences of gender with regard to VBS were not observed in studies enrolling nurses from other countries [36, 37]. The discrepancy may stem from cultural, regional, and departmental differences. In China, the increasing recognition and respect for male nurses in intensive care may enhance their professional self‐perception through affirmation from colleagues, patients, and the healthcare community [38, 39]. Chinese cultures have been characterized by a patriarchal structure [40], which not only empowers men in broader societal spheres (such as family governance, professional advancement, and public discourse) but also manifests in more nuanced ways within specialized fields such as healthcare, particularly in the context of nursing. In a predominantly female nursing field, male nurses may also attract more attention from supervisors and have greater opportunities for professional development, contributing to a positive self‐perception. Moreover, professional self‐perception may differ across different departments, which needs further research studies.

Employees with higher job titles empowered by skill variety and job autonomy may present with increased TWS and VBS [41, 42]. Interestingly, we observed that senior nurses and those in leadership positions exhibited lower levels of work‐related thriving and voice behavior compared to staff nurses. We hypothesize that increased work pressure and responsibility may contribute to this disparity. Middle‐level nurses are often burdened with managerial duties such as staff scheduling, resource allocation, and quality control, which lead to reduced thriving at work. They may adopt a more cautious approach, potentially leading to a reluctance to express their opinions to avoid potential errors or conflicts.

A research revealed that low household income led to reduced self‐perceptions of quality of life in young Iranian women [43]. Our findings indicated that average monthly household income, rather than the individual income of the surveyed nurses, positively correlated with professional self‐perception in the multivariable analysis. Previous studies also showed no association between individual monthly income and thriving at work for nurses [32, 44]. The study by Huang et al. reported a significant relationship between increased individual monthly income and high voice behavior, but the variable‐household monthly income was not included for analysis [45]. Moreover, we found that a one‐level increase in household income consistently elevated nurses’ professional self‐perception. Confucian values of family harmony and familialism position the family as the fundamental unit in Chinese society [19], where nurses’ professional self‐perception is tied to family stability and well‐being. Thus, average monthly household income, a reflection of fulfilling familial responsibilities through the collective efforts of family members, is positively correlated with nurses’ professional self‐perception, in contrast to individual income, which fails to capture this family‐centric value. This suggests that nursing managers could improve the self‐perception of nurses with low household incomes by increasing their salaries, though such measures may have limited effects on those with higher household incomes.

Other notable findings included that married nurses reported higher levels of work‐related thriving than single nurses. Previous research indicates that family‐supportive supervisory behaviors can enhance nurses’ thriving by mitigating work–family conflict and improving psychological well‐being [46]. A study by Duan JY et al. found that position was positively and significantly correlated with voice behavior, particularly in the Chinese context [47], aligning with our observations in critical care nursing. Furthermore, ICU experience emerged as an essential factor associated with voice behavior, which indicated that accumulated experience may lead to intensified voice behavior [48]. Another study demonstrated that older age is linked to an increased regulatory focus, especially in a prevention focus to minimize losses [49]. Our study consistently found that age significantly influenced both promotion and prevention focus among critical care nurses.

Interestingly, our results did not indicate that education level and weekly working hours were significantly associated with nurses’ professional self‐perception. Previous studies highlighted the important impact of education level on thriving at work and voice behavior [37, 50]. A plausible explanation is the small proportion of nurses with the master‐and‐above degree (1.2%), which may bias the estimation, although higher TWS, VBS, and AFS scores were observed in the master‐and‐above group (Supporting Figure S2). Previous studies have found that for Chinese emergency department nurses, prolonged working hours lead to a decrease in thriving at work [32], which is inconsistent with the results of our study. A potential reason was that, compared to the emergency department, ICU nursing has more stable work coping patterns, as well as stronger team support and standardized procedures. In contrast, the emergency department nurses require higher work intensity and emergency responsiveness, thus the impact of working hours is more significant. This inference requires direct comparison between the two departments for verification. Therefore, these negative results do not mean that we should ignore the importance of improving educational levels and ensuring reasonable working hours.

In summary, our study provided a large‐scale, geographically comprehensive insight into ICU nurses’ professional self‐perception in China and integrated TWS, VBS, and AFS as interconnected proxies for professional self‐perception and uncovered some novel findings shaped by Chinese culture (such as gender differences, influence of household income, and burden of managerial duties). The study shows that Chinese ICU nurses’ self‐perception is intertwined with family stability and social recognition and provides a culturally sensitive reference for future cross‐cultural comparisons.

### 4.1. Implication for Nursing Management

The results of this study have important implications for managers and leaders seeking to improve nurses’ professional self‐perception.

1. Targeted mentorship programs: Especially for young, single, and less experienced nurses, a stratified mentorship program should be implemented: Assign a high‐seniority ICU nurse as a mentor to junior nurses. Mentorship content should combine clinical skill coaching (e.g., hands‐on training for critical procedures such as mechanical ventilation) to boost competence and psychological counseling to enhance resilience. In addition, mentors should encourage junior nurses to voice clinical concerns (e.g., suggesting workflow improvements) in team meetings, with the mentor advocating for their ideas initially. The program includes 1‐h weekly one‐on‐one sessions and monthly group workshops, where successful case studies highlighting junior nurses’ contributions are shared, to reinforce their positive professional self‐perception.
2. Resilience training: Resilience training is tailored to middle‐level professional title nurses. Middle‐level nurses show reduced professional self‐perception, which is likely due to heavy managerial workloads (e.g., staff scheduling and quality control) and reduced clinical autonomy. Therefore, a resilience training program focused on stress mitigation and role reorientation is needed. Middle‐level nurses should be trained to delegate noncore administrative tasks (e.g., routine report filing) to junior nurses or dedicated nursing assistants, using a “task priority matrix” to reduce time spent on low‐value work, as this alleviates burnout and frees up time for clinical practice. Meanwhile, the training program integrates regulatory focus‐aligned exercises to elevate their focus from “avoiding errors” (prevention focus) and “achieving impact” (promotion focus), enhancing their sense of professional value.
3. Family‐supportive policies: Work performance rewards and targeted subsidies should be allocated preferentially to nurses from low‐income households to provide economic support and improve household economic stability. Partners with hospitals and local community centers were motivated to offer free/low‐cost family services (e.g., after‐school care for nurses’ children and elderly caregiver training for nurses’ family members) to reduce nurses’ out‐of‐work care burdens.
4. Workload reduction and voice empowerment: Integrated electronic health record (EHR) systems could be used to streamline documentation and reduce non‐nursing tasks. This procedure frees up time for nurses to engage in proactive communication (e.g., discussing treatment plans with physicians), boosting professional self‐perception. Voice channels should be established with a “nurse suggestion committee,” where nurses (regardless of position) can propose workflow or policy improvements (e.g., optimizing shift patterns). This validates nurses’ contributions and enhances their sense of professional self‐perception.
5. Gender‐sensitive support: Conduct antistereotype training for hospital administrators and physicians to raise awareness of implicit gender biases (e.g., nursing is a “female” job with limited advancement), offer “work–life balance counseling” for female nurses during life transitions (e.g., pregnancy and postpartum return to work), and provide flexible shift options (e.g., reduced hours after postpartum) to avoid career interruptions, supporting their sense of continuity and professional growth.

These recommendations target our study’s identified risk factors and link to the three dimensions of professional self‐perception (TWS, VBS, and AFS). By combining individual‐level support (mentorship and training), family‐level assistance (subsidies and services), and organization‐level policy adjustments (workload cuts and voice channels), nursing managers can implement interventions to enhance ICU nurses’ professional self‐perception.

**FIGURE 4:**
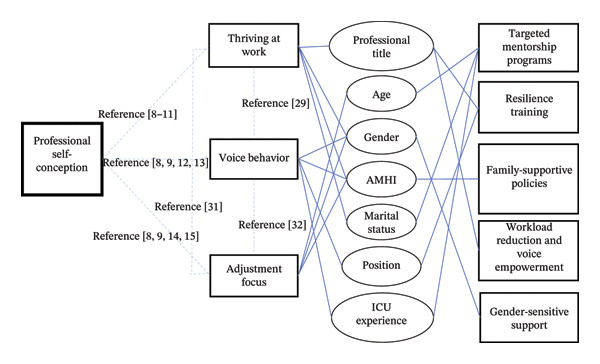
The significant findings, holistic framework of the professional self‐perception, and corresponding managerial measures (dotted lines denote structural connections of the self‐perception, and solid lines indicate the influencing factors and their corresponding managerial measures). AMHI: average monthly household income.

In summary, Figure 4 synthesizes the holistic framework of professional self‐perception, significant findings, and the corresponding managerial measures.

### 4.2. Limitations

This study has several limitations that should be acknowledged. First, professional self‐perception among ICU nurses is a complex multidimensional construct, and some of its dimensions were not measured herein. Instead, this study used three proxy dimensions to reflect this concept rather than directly assessing it.

Second, as a cross‐sectional study, it only captures the current state and does not allow for causal inferences and analyzing temporal relationships. Future longitudinal studies could provide insights into how professional self‐perception changes among ICU nurses and how targeted interventions such as family‐supportive policies or enriched ICU experiences improve professional self‐perception.

Third, while we employed a combination of stratified and convenience sampling, this approach may have introduced certain selection biases. In addition, the demographic characteristics of our sample, such as age, gender, and years of experience, may not precisely reflect the national profile of ICU nurses, thus the potential over‐ or underrepresentation may influence the generalizability of our findings. Future research employing randomized sampling and larger, nationally representative cohorts is warranted to mitigate confounding and selection biases and enhance the generalizability of the findings.

Fourth, while we adjusted for a comprehensive set of sociodemographic and occupational factors, the potential for unmeasured confounding, such as leadership styles or access to psychological support services, remains. Therefore, our findings should be interpreted as identifying robust associations rather than establishing causal relationships. Future studies incorporating those factors may enrich our findings.

Lastly, caution should be taken when generalizing our findings to settings outside the ICU or to different cultural contexts and countries.

## 5. Conclusion

ICU nurses in China exhibit a moderate level of professional self‐perception. Factors associated with lower self‐perception include youth, single marital status, female gender, low household income, middle‐level professional title, lower occupational position, and limited ICU experience. To enhance professional self‐perception, nursing managers may consider targeted interventions for these specific demographic and professional groups. Importantly, these findings are specific to ICU nurses in the Chinese healthcare context, and thus may not be generalizable to other nursing populations (e.g., general ward nurses and community health nurses) or healthcare contexts outside China. The study’s cross‐sectional design and the potential bias further limit causal inferences and broader generalization, and future longitudinal studies should be conducted to explore temporal changes in professional self‐perception and validate context‐specific interventions.

## Author Contributions

Xin Liu: conceptualization, project administration, and writing–original draft.

Xuan Zhang: writing–original draft, methodology, and data curation.

Zewen Pan: writing–original draft, methodology, and data curation.

Yu Xu, Zhongwen Sun, Haixia Tang, and Lei Chen: investigation and supervision.

Jianhua Tang, Zheng Jia, Yue Huang, and Ying Peng: investigation and data curation.

Hanfeng Zhang: project administration, supervision, and writing–review and editing.

Zhenjun Liu: conceptualization, formal analysis, visualization, and writing– review and editing.

## Funding

The work was supported by the Key Clinical Construction Project of Sichuan Province (Grant no. YB2022009).

## Ethics Statement

The study protocol was approved by the Ethics Committee for Medical Research of Sichuan Cancer Hospital (SCCHEC‐02‐2024‐216).

## Conflicts of Interest

The authors declare no conflicts of interest.

## Supporting Information

Supporting Figure 1: Provincial and regional distribution of the study. (a) Provincial distribution covered by the study and the regional response rates. (b) Regional distribution of ICU nurses participating in the study.

Supporting Figure 2: Comparison of nonsignificant sociodemographic factors related to professional self‐perception in the violin plots. (a) TWS. (b) VBS. (c) AFS. TWS: Thriving at Work Scale; VBS: Voice Behavior Scale; AFS: Adjustment Focus Scale. The wider parts of the violin represent areas where the data are more densely concentrated, the line inside the box represents the median value, and the box represents the interquartile range. The whiskers extend from the box and represent the range of the data; outliers are represented with solid dots in whiskers, and subtransparent dots represent data distribution with some jitters.

Supporting Figure 3: Sensitivity analysis of gender differences related to professional self‐perception in the violin plots. TWS: Thriving at Work Scale; VBS: Voice Behavior Scale; AFS: Adjustment Focus Scale. The wider parts of the violin represent areas where the data are more densely concentrated, the line inside the box represents the median value, and the box represents the interquartile range. The whiskers extend from the box and represent the range of the data; outliers are represented with solid dots in whiskers, and subtransparent dots represent data distribution with some jitters. ^∗^ *p* < 0.05, ^∗∗^ *p* < 0.01, and ^∗∗∗^ *p* < 0.001.

## Supporting information


**Supporting Information** Additional supporting information can be found online in the Supporting Information section.

## Data Availability

The data of this study are available from the corresponding author upon reasonable request.
