# Supplementary material for: Professional Self‐Perception Among Critical Care Nurses in China: A Nationwide Cross‐Sectional Survey
Source: J Nurs Manag. 2026 Mar 13;2026:6657778. doi: 10.1155/jonm/6657778 (PMC13140179; doi:10.1155/jonm/6657778)
Supplement: Supplementary file 1 — Supporting Information Additional supporting information can be found online in the Supporting Information section. [file JONM-2026-6657778-s001.zip › Supplementary materials.docx]

**The results of univariable analysis**

We found significant differences between gender, marital status, professional title, average monthly income, and average monthly household income for the TWS (all p < 0.05).

Significant differences were observed in terms of gender, marital status , professional title , average monthly income, average monthly household income, age, ICU experience, fertility status , position , and nurse specialist training pertinent to VBS scores (all p < 0.05).

In AFS scores, we found significant differences between gender , marital status, age , average monthly income , average monthly household income, nurse specialist training, fertility status, and position (all p<0.05). These results are depicted in Figure 2.

Interestingly, our results did not indicated that education level and weekly working hours significantly associated with nurses’professional self-perception (both p>0.05). Variables without significant comparison differences are illustrated in Supplementary Figure 2.

**The results of multivariable analysis**

MOLR analysis revealed a significant association between gender and thriving at work (adjusted OR, 95% CI: 2.01, 1.32-3.07, p < 0.001), voice behavior (adjusted OR, 95% CI: 2.15, 1.39-3.33, p < 0.001), and adjustment focus (adjusted OR, 95% CI: 1.74, 1.14-2.66, p = 0.002).

Average monthly household income was found to be a significant factor influencing thriving at work (adjusted OR, 95% CI: 1.51, 1.19-1.92, p < 0.001), voice behavior (adjusted OR, 95% CI: 1.35, 1.05-1.74, p = 0.018), and adjustment focus (adjusted OR, 95% CI: 1.47, 1.16-1.86, p = 0.002). Interestingly, professional title was inversely associated with TWS (adjusted OR, 95% CI: 0.73, 0.55-0.96, p = 0.027) and VBS (adjusted OR, 95% CI: 0.64, 0.47-0.88, p = 0.006).

Additionally, marital status was independently associated with thriving at work (adjusted OR, 95% CI: 1.52, 1.06-2.19, p = 0.022). ICU experience (adjusted OR, 95% CI: 1.28, 1.04-1.59, p = 0.021) and position (adjusted OR, 95% CI: 1.63, 1.22-2.18, p = 0.001) were significantly associated with voice behavior. Age was independently associated with adjustment focus (adjusted OR, 95% CI: 1.52, 1.06-2.19, p = 0.022). The results are shown in Table 2
