# Supplementary figures and images for: Professional Self‐Perception Among Critical Care Nurses in China: A Nationwide Cross‐Sectional Survey
Source: J Nurs Manag. 2026 Mar 13;2026:6657778. doi: 10.1155/jonm/6657778 (PMC13140179; doi:10.1155/jonm/6657778)

(a)

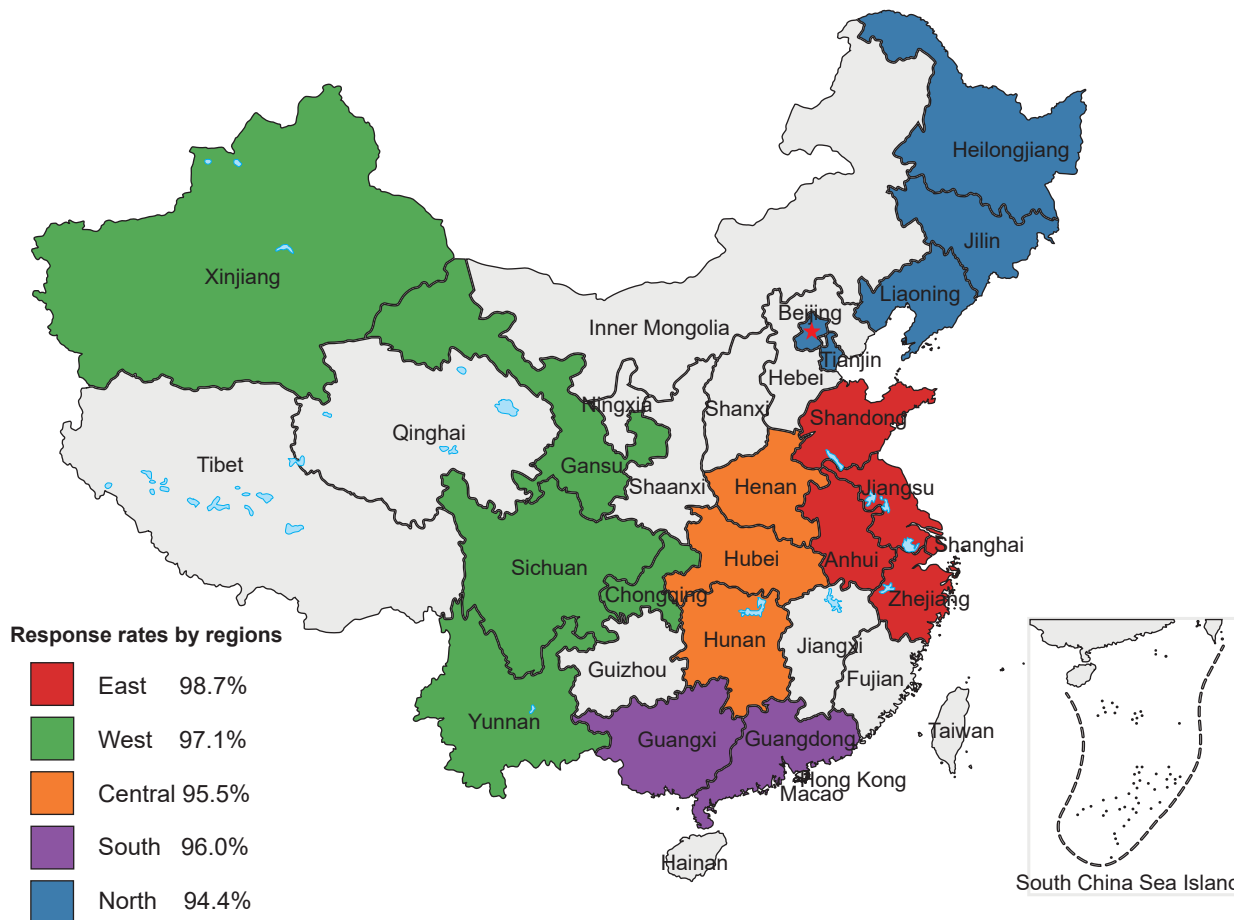

(b)

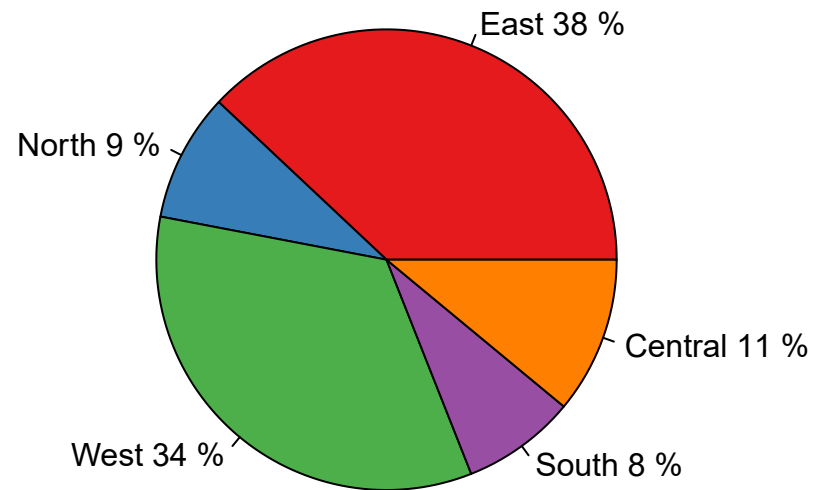

Supplement: Supplementary file 1 — Supporting Information Additional supporting information can be found online in the Supporting Information section. [file JONM-2026-6657778-s001.zip › figure s1.pdf]

(a) TWS

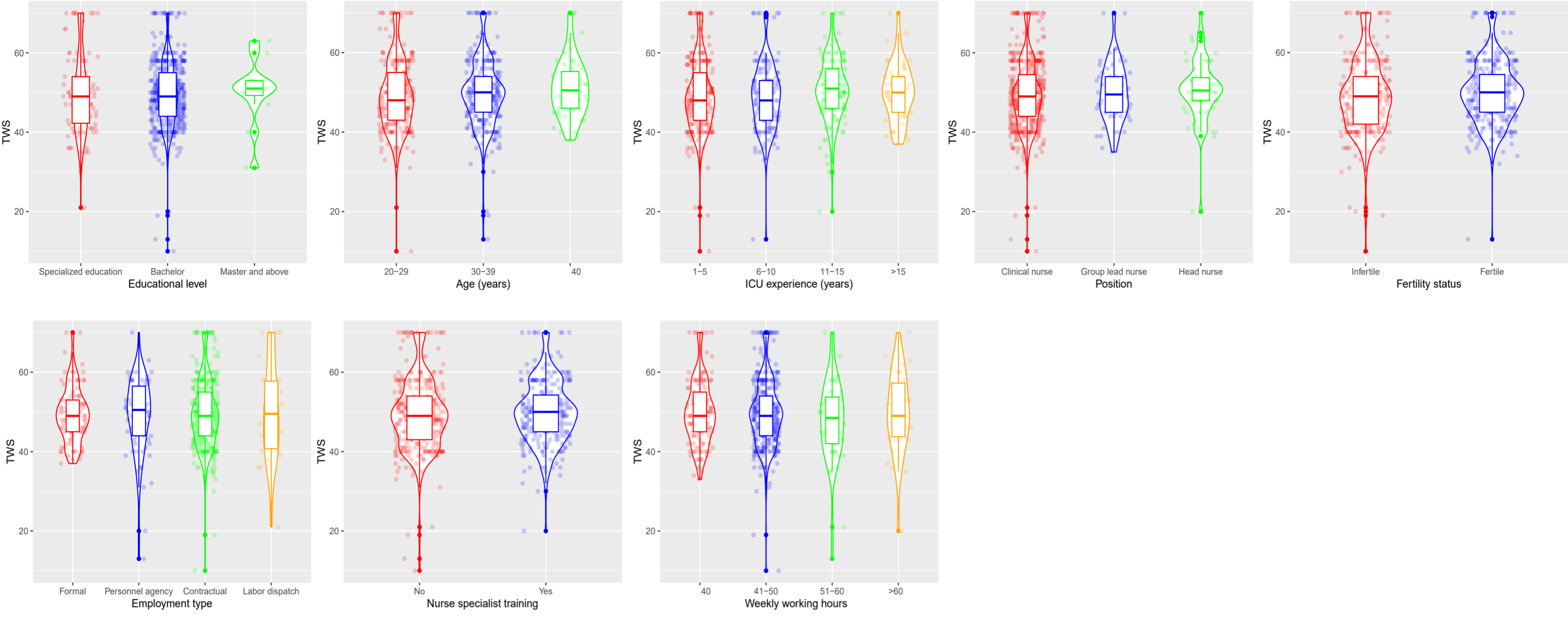

(b) VBS

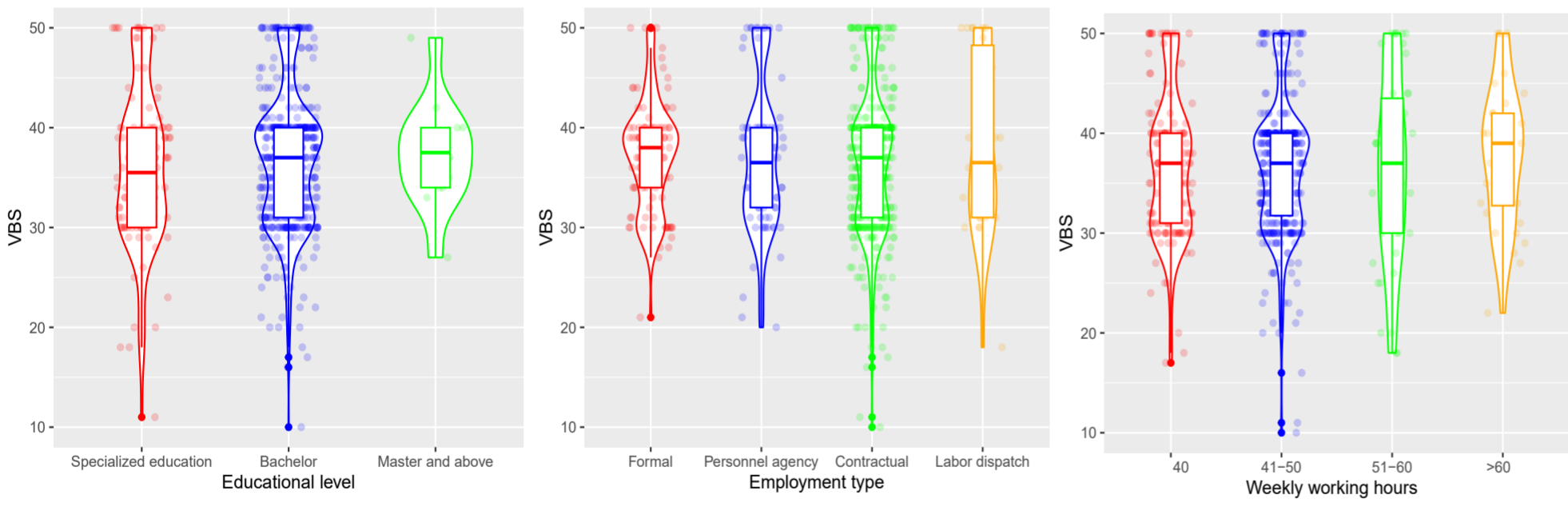

(c) AFS

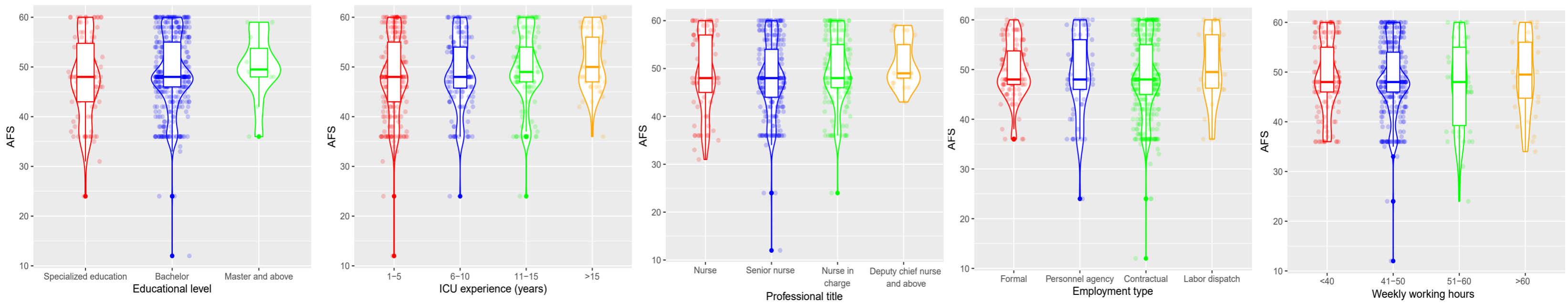

Supplement: Supplementary file 1 — Supporting Information Additional supporting information can be found online in the Supporting Information section. [file JONM-2026-6657778-s001.zip › figure s2.pdf]

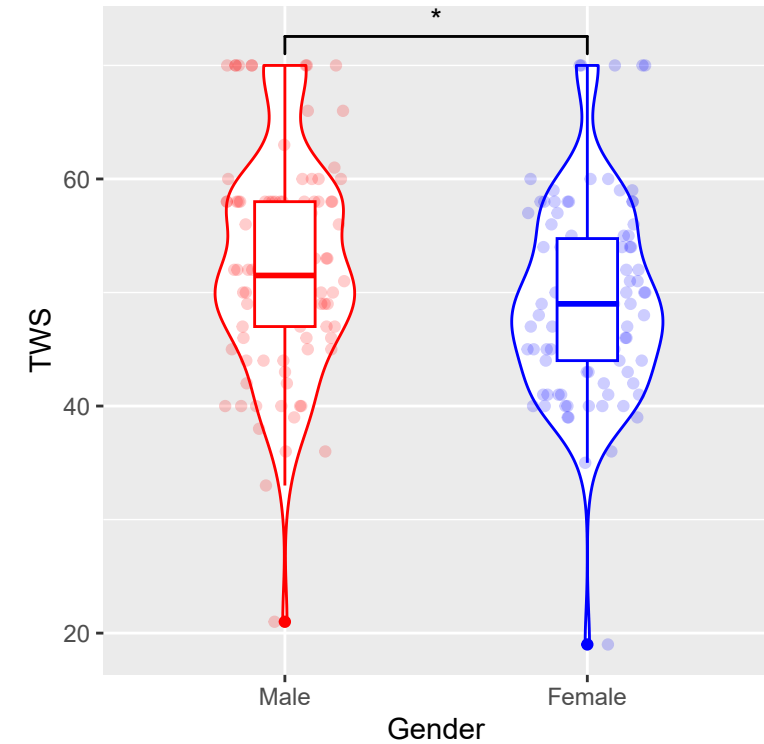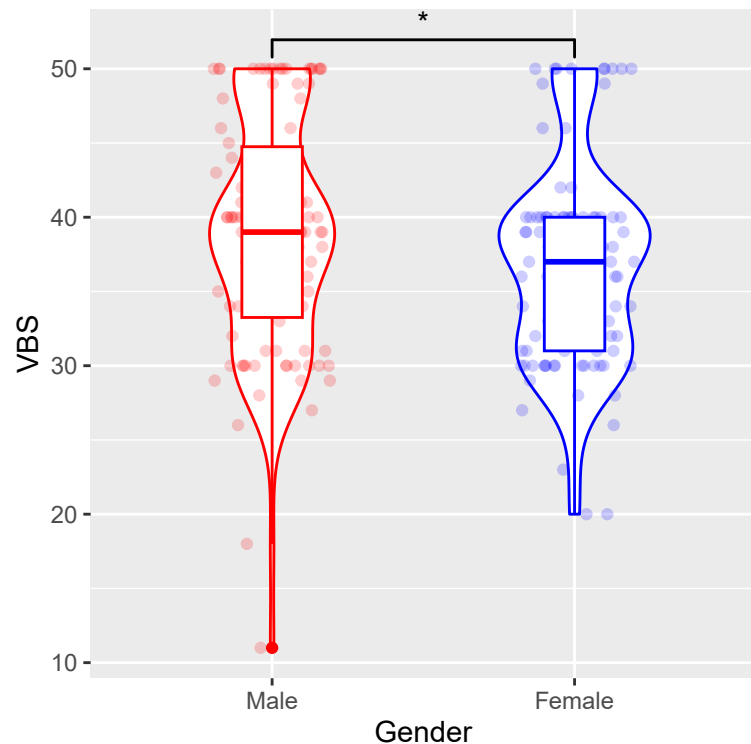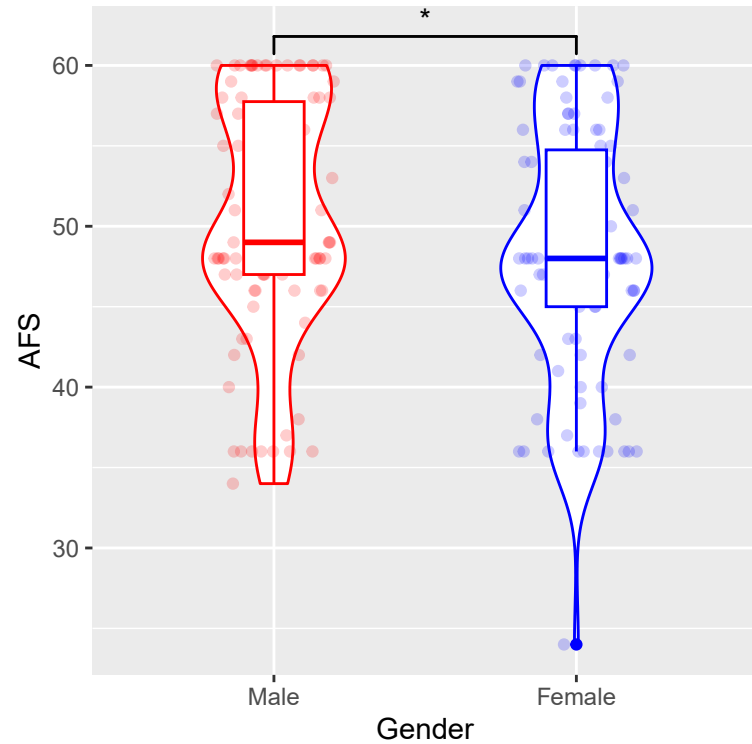

Supplement: Supplementary file 1 — Supporting Information Additional supporting information can be found online in the Supporting Information section. [file JONM-2026-6657778-s001.zip › figure s3.pdf]
